# Undergraduate research experiences: mentoring, awareness, and perceptions—a case study at a Hispanic-serving institution

**DOI:** 10.1186/s40594-018-0105-8

**Published:** 2018-04-02

**Authors:** Laura Rodríguez Amaya, Tania Betancourt, Kristina Henry Collins, Orlando Hinojosa, Carlos Corona

**Affiliations:** 1 LBJ Institute for STEM Education and Research, Texas State University, San Marcos, TX USA; 2 Materials Science, Engineering, and Commercialization Program, Texas State University, San Marcos, TX USA; 3 Department of Chemistry and Biochemistry, Texas State University, San Marcos, TX USA; 4 Curriculum and Instruction Core Faculty for Talent Development, Texas State University, San Marcos, TX USA

**Keywords:** Research awareness, Research perceptions, Undergraduate research experiences, Mentorship, Minorities in STEM, Hispanic-serving institution

## Abstract

**Background:**

Undergraduate research experiences (UREs) have been proposed as means to increase the retention and engagement of minority—and more specifically Hispanic—college students in science, technology, engineering, and mathematics (STEM) majors. This study explores the impact of student characteristics such as gender, classification, ethnicity, and first-generation status on UREs of STEM students through four specific constructs that current literature deem particularly important: (1) research experiences, (2) mentoring experiences, (3) awareness of research opportunities and activities, and (4) perceptions on research. These constructs are here forth referred to as Experiences, Mentoring, Awareness, and Perceptions. The study was conducted at a Hispanic-serving institution (HSI) in Texas, United States (U.S.), where the overall increase in enrollment has been driven by growth in Hispanic student numbers, reflecting the demographic shift of the state and the nation.

**Results:**

Participants were recruited to be part of a STEM open house. Thirty-five students participated in the Undergraduate Research Experiences: Mentoring, Awareness, and Perceptions Survey (URE MAPS). This exploratory case study sought to look at student characteristics such as gender, classification, ethnicity, and first-generation status as predictors of UREs. Results show that classification and ethnicity student characteristics are statistically significant predictors of UREs. Although gender and first-generation status regression analysis did not show statistically significant results, crosstabulations looking at correlation among variables yield interesting results. Seven percent of the female respondents responded that they “somewhat agree” with the statement that research is a lonely activity in comparison with 23% of males. The majority (60%) of all respondents who “strongly agreed” with the statement that “research is only for future scientists” were Hispanic, indicating a need to clarify such misconceptions to encourage Hispanic student participation. Most self-identified first-generation participants, of whom 80% were female, reported awareness of faculty research activities, again pointing out gender as an important factor among students’ relationship with their professors. Although less than 23% of students noted current participation in mentorship, most of those did report positive impact of this relationship on their attitude and perspective toward their major.

**Conclusions:**

Despite the small sample size and inherent bias in the characteristics of the STEM open house participants, regression analysis informed by crosstabs analysis revealed some important findings. The research suggested higher-than-expected awareness of Latinos and first-generation students of institutional research activities; however, this awareness has not translated in engagement in research activities. The data also indicates the critical need for high-impact UREs and mentorship relationships, as well as for efforts to battle student preconceptions of who can benefit from such experiences. Although this case study focused on LatinX students (LatinX is a gender-neutral term for people of Latin American heritage used in the U.S.) in the U.S., retention of historically underrepresented students in STEM disciplines is a concern shared by many countries around the world. The successful recruitment, retention, and eventual success of students in STEM degrees depend greatly on the type of pathways and support that are offered. UREs might be one of those pathways.

## Background

To remain or become globally competitive, many countries have developed strategic national science, technology, engineering, and mathematics (STEM) policy frameworks. STEM-strong countries are diverse economically, politically, and socially. Their STEM policy frameworks include a variety of services and activities designed to address factors affecting underrepresented minority students’ interest, motivation, and skills in STEM (Hulme and De Wilde 2014). A common feature shared among these initiatives is an emphasis on making STEM more engaging, practical, and student-centered. The ultimate goal of these programs is to diversity the STEM profession by attracting historically underrepresented minorities (women, indigenous/ethnic/cultural groups) and sustain their persistence in STEM fields (Christie et al. 2017).

According to Colby and Ortman (2015), the Two or More Races is the fastest-growing population in the U.S. with the Asian population coming in a close second. The Hispanic (in this paper, the terms Hispanics and Latinos are used interchangeably) population is projected to be the third fastest growing. The Hispanic population is projected to increase from the reported 55 million in 2014 to 119 million by 2060, an increase of 116%. By 2060, 29% of the U.S. is projected to be Hispanic, representing more than one quarter of the total population (Colby and Ortman 2015). Other minority groups’ populations such as African Americans are expected to remain constant, seeing only modest growth in the next 40 years.

While minority, and more specifically Hispanic, student enrollment in higher education continues to grow, diversification of STEM professions remains a challenge. Martinez Ortiz and Sriraman (2015) suggest that some of the best approaches to pursue in diversifying the pipeline of STEM professionals are to focus on retaining undergraduate students of diverse backgrounds who are currently in STEM fields of study and to support them to successfully graduate. Undergraduate research experiences (UREs) have shown positive effects in increasing students’ engagement in their undergraduate studies, an increase in understanding of their field of study; an increase in practical skills such as problem solving, communication, and information synthesis; and an increase in interest in graduate school (Haeger et al. 2015; Willis et al. 2013). All of these are important aspects of the education experience toward successful completion of a bachelor’s degree.

UREs are believed to increase persistence in STEM degree programs and incite interest in higher degrees (Brewer and Smith 2011; Graham et al. 2013; Russell et al. 2007; Zydney et al. 2002). Russell et al.’s (2007) study of 15,000 students indicates that UREs “clarify students’ interest in research and encourage students who hadn’t anticipated graduate studies to alter direction toward a Ph.D.” There are significant benefits of UREs beyond developing expertise in a specific academic area that are as important for employers as graduate programs. These include the development of team ethics, problem-solving, and communication skills and a better understanding of their career path (Zydney et al. 2002). Almost 20 years ago, a report from the National Science Foundation (NSF 1989) stated “it is clear that the academic scientific community regards the involvement of undergraduate student majors in meaningful research and related scholarly activity with faculty members as one of the most powerful instructional tools.”

In order to engage students in meaningful research, faculty involvement is critical. Faculty mentoring in UREs can differentiate research experiences from typical labs and strengthen student impact (Fechheimer et al. 2011; Linn et al. 2015; Taraban and Logue 2012; Thiry et al. 2011). Not only mentoring provides the necessary guidance to students in their learning but also an effective faculty mentor also assists students with their self-identity development as scientists (Linn et al. 2015; Munawar 2015). Faculty mentoring in STEM can enhance positive student development and result in increased student interest in graduate school, engagement in their undergraduate studies, understanding of their field of study, and an increase in practical skills (Hunter et al. 2007). Undergraduate researchers from underrepresented groups such as African American and Latina/o students reported higher learning gains than comparison students when they participated in UREs (Linn et al. 2015; Munawar 2015). Research supports that underrepresented students tend to benefit the most from faculty mentoring (Linn et al. 2015).

The study was conducted at a Hispanic-serving institution (HSI) in the state of Texas, U.S., where the increase in overall enrollment is driven by Hispanic student enrollment, reflecting the demographic shift of the state and the nation. HSIs in the U.S. are defined as not-for-profit institutions of higher learning with a full-time equivalent (FTE) undergraduate student enrollment that is at least 25% Hispanic. The study site presents a unique opportunity to focus the study on LatinX students (LatinX is a gender-neutral term for people of Latin American heritage used in the U.S.). As Haeger et al. (2015) state, there is only a limited number of studies on the participation of LatinX and other minority students in UREs. This study explores the UREs of undergraduate STEM students and the predictors of participation based on student characteristics such as ethnicity, gender, first-generation status (first-generation college student is defined as a student whose parent(s)/legal guardian(s) have not completed a bachelor’s degree), and classification. To this end, the following is the research question pursued in this study:

1. Do ethnicity, gender, first-generation status, and classification predict the following UREs:
  - Research experiences
  - Mentoring experiences
  - Awareness of research opportunities and activities
  - Perceptions on research

### Background on the four constructs of UREs selected for this study

#### Awareness and perception of research activities and their benefits

Research shows that a majority of students lack awareness of research opportunities being conducted within their own programs and the university at large. In a study conducted by Munawar (2015) aimed to determine research awareness, perceptions of competency, and research motivations in 20 first- and second-year bachelor of medicine/bachelor of surgery students at Shalamar Medical and Dental College at Lahore in Pakistan, only 10% of surveyed students were familiar with research opportunities at their institution. Of those with the awareness of URE opportunities, preconceived, false stereotypes such as the belief that research entails working in socially isolated environments can create barriers that deter students from participating. This and other misconceptions about the roles of a scientist in research can be dispelled through effective UREs (Adedokun and Burgess 2011). UREs can also provide opportunities to foster and develop an extensive list of benefits to participating faculty and students. Gains commonly seen include increased awareness, increased clarity of future goals in STEM careers, gained knowledge of how to work like a scientist, enhanced graduate school readiness, and clarified perceptions (Seymour et al. 2004; Hunter et al. 2007). Research also shows that students perceived improvement in communication skills, conceptual and analytical thinking, understanding of scientific work, and confidence in problem solving (Lopatto 2003).

#### Effect of research experiences on motivation and retention in STEM

Studies have shown that academic and professional motivation to obtain a STEM degree does not arise from a single aspect, but rather multiple aspects including class experience, science identity, and research intentions (Smith et al. 2014). Implementation of UREs provide spaces and opportunities for these multiple components to improve student motivation within STEM. Students that participated in UREs claimed they decided to become involved in research as a way to build their resume, gain familiarity with faculty for future references, and obtain experience that future employers may like to see in a graduate (Tykot et al. 2014). Nagda et al. (1998) suggested that research involvement is “most effective in promoting the retention of students at greater risk for college attrition.” When students participate in research, they are motivated to perform better in class, stay in school, and engage in the scientific community.

#### Effect of research participation on student graduate school and career persistence

UREs influence students’ decisions about their future career and educational opportunities. They increase awareness of STEM career options, provide career clarification, and enhance students’ cognitive and personal skills alongside their professional credentials (Adedokun et al. 2012; Adedokun et al. 2013). The overwhelming majority of undergraduate researchers reported that their research experience increased student engagement at the undergraduate level, either sustained or increased their interest in post-graduate education, and fostered an increased understanding of their field of study (Willis et al. 2013). Hathaway et al. (2002) found that undergraduate research participants were significantly more likely to pursue graduate education and additional research activity. These scholastic gains, combined with UREs, also improve career clarification and options for STEM students. Additionally, students of color who participated in undergraduate research were significantly more likely to pursue graduate education than students of color who did not participate in undergraduate research (Hathaway et al. 2002).

#### Undergraduate research as a means to establish mentorship relationships

The literature on the contexts and benefits of mentoring are well documented (Kochan and Pascarelli 2003). Mentoring, offering psychosocial support that is absent from academic coaching and advising alone, has significant impact on students, and especially underrepresented students in STEM (Winkle-Wagner et al. 2010; Collins 2013), in terms of positive self-concept, academic success, and persistence. Mentees often report mentors as the biggest influence in career selection, often times choosing a very similar career path as the mentor. UREs help create a space for STEM students to engage in meaningful relationships with faculty mentors. The role of the faculty and their perceptions are critical in the learning outcomes and impact of UREs.

Faculty mentoring in STEM, when clearly articulated, can enhance positive URE effects with an increase in practical skills, problem solving, communication, and information synthesis (Willis et al. 2013). Additionally, Hunter et al. (2007) reported that respondents involved in graduate school or research-related careers were much more likely to have reported a faculty member playing an important role in their choice. While underrepresented students tend to benefit the most from mentoring, the literature suggests a “conundrum between mentor availability and mentor impact” that is created by faculty presumptions of their students and student misconceptions of the mentoring relationship (Linn et al. 2015).

## Methods

**Fig. 1:**
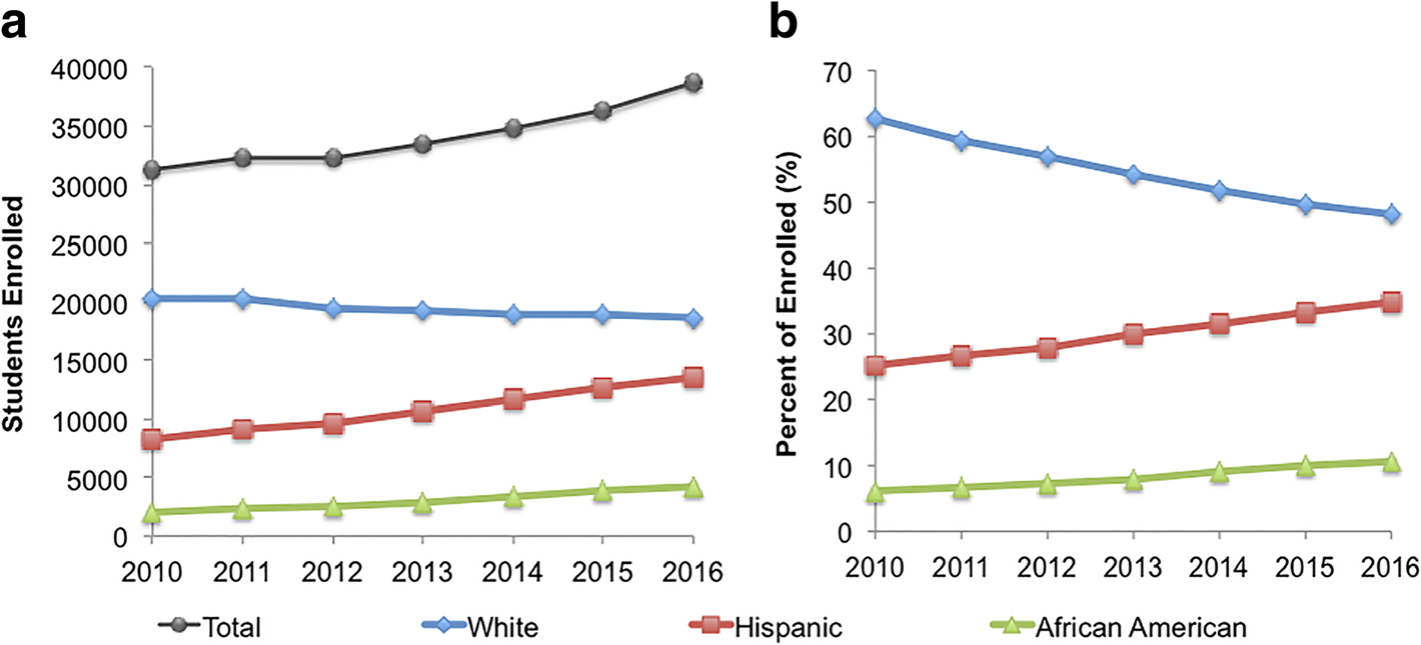
Student enrollment demographics. **a** Student enrollment at university in the period from 2010 to 2016. **b** Enrollment percentages as a function of demographic groups

This study was conducted during an undergraduate STEM open house event at a HSI in the state of Texas, U.S. Texas is one of the fastest-growing states in the nation. In 2015, Hispanics represented 39% of the total population in Texas, and it was projected that the Hispanic population in Texas will double in the next 20 years (Parada et al. 2016). At the HSI selected as the study site, the Hispanic student population of 35% closely resembles that of Texas’ Hispanic population of 39% (Parada et al. 2016). The Black student population of 11% at the university also contributes to the growing representation of minority students at this university. Figure 1 illustrates that the overall increase in enrollment over the past 6 years is driven by an increase in Hispanic student enrollment, reflecting the demographic shift of the state. Figures for the College of Science and Engineering (COSE) at this HSI closely follow this trend, with the current Hispanic and Black student enrollment at 31 and 10%, respectively.

### STEM student recruitment

Students were recruited into the research project as part of an undergraduate STEM open house event. The target audience was freshman and sophomore STEM majors. Flyers around campus advertised the undergraduate STEM open house (here thereafter referred as the open house or event). In addition, electronic versions of the flyers along with contact information were emailed by COSE to all STEM majors with freshmen and sophomore classification and posted on the university’s social media accounts. The event was also communicated to most of the COSE faculty so that they could provide the information and the flyer to their students in class, by email, and/or through the online class management system. Interested students were directed to complete a web-based survey to indicate their interest.

**Fig. 2:**
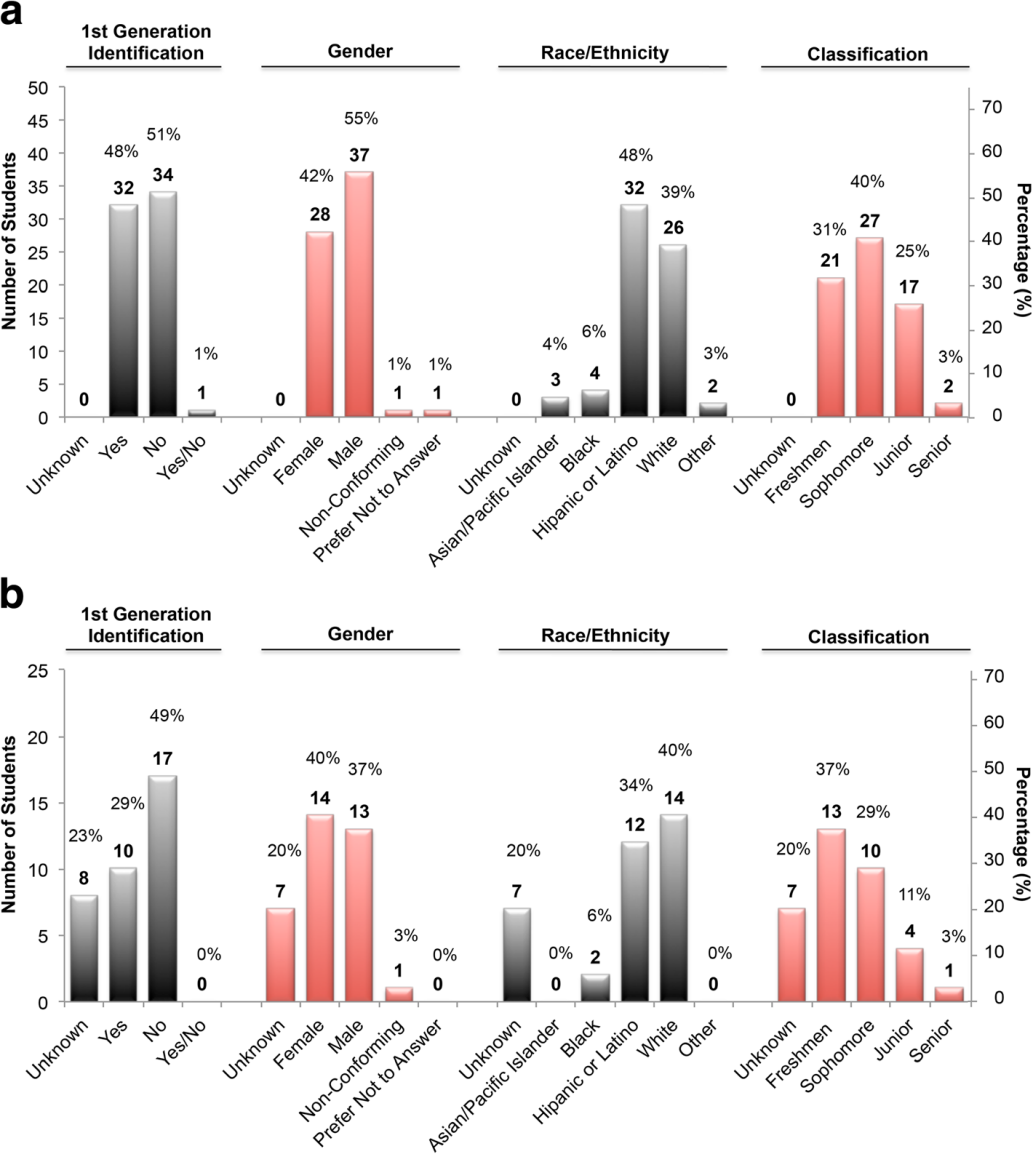
Demographic details of student applicants and survey participants. **a** Numbers and percentages of student applicants (*n*_total_ = 67) as a function of identification as first-generation students, gender, race/ethnicity, and classification. **b** Numbers and percentages of student participants (*n*_total_ = 35) as a function of identification as first-generation students, gender, race/ethnicity, and classification. Left and right axes represent the number and percentage of students in each category, respectively. Numbers on top of bars represent the number of students in each category

**Fig. 3:**
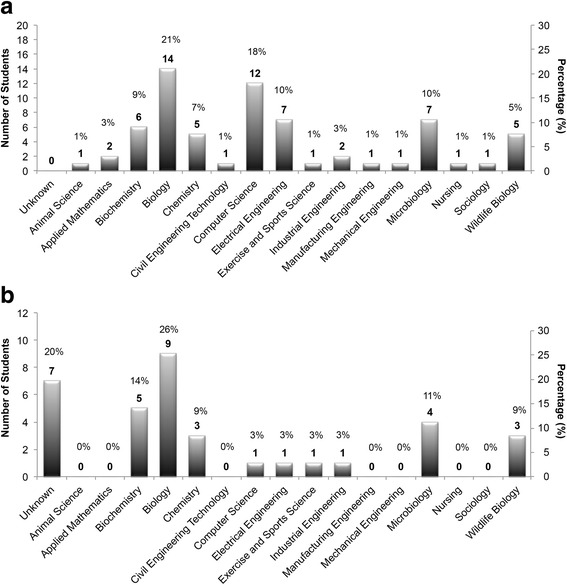
Distribution of student majors. **a** Numbers and percentages of student applicants in each major (*n*_total_ = 67). **b** Numbers and percentages of students in each major who participated in event survey (*n*_total_ = 35). Left and right axes represent the number and percentage of students in each category, respectively. Numbers on top of bars represent the number of students in each category

Most (96%) of the students that applied to participate in the event had not been involved in research. Sixty-three out of 67 students (94%) were accepted to participate. The students that were not invited to participate were either already involved in research (3/4), not majoring in STEM fields (1/4), or of junior classification (1/4). Figures 2 and 3 detail the characteristics of the students that applied to the event.

### Survey design

The Undergraduate Research Experiences: Mentoring, Awareness, and Perceptions Survey (URE MAPS) tool included both closed-ended and open-ended questions to allow for a more complete understanding of the four constructs stated above. Participants answered 22 questions on a Likert scale out of 30 questions. Three binary (yes/no) questions and five open-ended questions composed the remaining eight questions. Questions were organized by construct (refer to Table 1 for detailed information).

**Table 1:** Undergraduate Research Experiences: Mentoring Awareness, and Perceptions Survey (URE MAPS)

Construct (response variable)	Question	Indicators
Experiences	*Faculty in this section refers to professors, researchers, and/or scientists at the university.* During your time at the university, how often do you do the following? *[Never, sometimes, about half of the time, most of the time, always]*	
	1_1: Talk about possible research opportunities with a faculty at the university	E_Talk_University Faculty
	1_2: Talk about possible research opportunities with someone outside the university	E_Talk_Faculty Outside
	1_3: Work with a faculty member on an out-of-class research-based project other than coursework	E_Work_University Faculty
	1_4: Work with someone outside the university on a research-based project other than coursework	E_Work_Outside
	1_5: Discuss your career aspirations with a faculty member at the university	E_Aspirations_University Faculty
	1_6: Visit a research lab at the university	E_Visit University Lab
	1_7: Visit a research lab outside the university	E_Visit Lab Outside
Mentoring	*For the questions below use the following definition for a faculty mentor. A faculty mentor serves as a guide to the institution and its culture, as a resource, as a research advisor, and as a career guide. It can be assigned to you as part of a formal program or you can seek out this relationship independently.* Have you done or plan to do the following before you graduate? *[yes/no]*	
	2_1: Work with a faculty member on a research project	M_Work with Faculty
	2_2: Ask a faculty member that engages in active research to be your mentor	M_Ask Faculty
	2_3: Join a mentoring program that includes research as component of the program	M_Join Program
	Do you have a mentor at the university?	Open-ended
	2_4: What mentoring experience has been the most significant for you at the university, and what has been the most disappointing?	Open-ended
	2_5: Has mentoring positively affected your attitude and perspective toward your major. If yes, how so?	Open-ended
Awareness	During the current academic year, about how often were you aware of the following? *[Never, sometimes, about half of the time, most of the time, always]*	
	3_1: Ongoing research performed here at the University	A_R_University
	3_2: Ongoing research performed in the department of your selected major (e.g. biology, physics, engineering, etc.)	A_R_Department
	3_3: Ongoing research performed by your professors	A_R_Your Professors
	3_4: Publications by professors at university	A_P_ University Professors
	3_5: Publications by your professors within your major	A_P_Major Professors
	3_6: Undergraduate research conferences/exhibitions opportunities at university	A_UG R_Conferences at University
	3_7: Undergraduate research presentations/ exhibitions opportunities in your department	A_UG R_Conferences Department
	3_8: Undergraduate research conferences outside university or within the larger field of your major	A_UG R_Outside University
Perceptions	To what extent do you agree or disagree with the following statements about research? *[strongly disagree, somewhat disagree, neither disagree nor agree, somewhat agree, strongly agree]*	
	4_1: Research is a relevant activity for me as an undergraduate student	P_R_Relevant
	4_2: Research is only for students that want to work in a lab	P_R_ Want to Work in a Lab
	4_3: Research is only for students that want to be scientists	P_R_Only for Future Scientists
	4_4: Research can help me learn more about my intended career field	P_R_Learn about Field
	4_5: Doing research is a waste of my time	P_R_Waste Time
	4_6: Doing research can help me develop important skills for my adult life	P_R_Skills
	4_7: Doing research is a lonely activity	P_R_Lonely

### Data analysis

#### Multivariate analysis

Basic statistical analysis was conducted to inform the multivariate analysis selection process. Analysis of some individual indicators selected for this study revealed non-parametric and non-linear relationships. To acquire acceptable results, non-linear and non-parametric approaches were needed to explore the structure of the data.

First, the indicators for the four constructs of URE MAPS went through two multivariate analyses: non-linear principal component analysis (NLPCA) and Cronbach’s coefficient alpha. For the analysis, the CATPCA (extended or categorical principal component analysis) approach in the Statistical Package for the Social Sciences (SPSS) was selected for its suitability to handle different scale levels. CATPCA is often used for data reduction. However, in this study, it is used as a tool to assess the reliability of the indicators to measure the same underlying construct and to understand better the dimensionality of the constructs. The goal of principal component analysis (PCA) is to reveal how different variables change in relation to each other and how they are associated (Nardo et al. 2008). Data reduction, in this case by indicator selection, is only considered in the components where instability is suspected in the CATPCA solution and corrected by the exclusion of variables.

Second, the use of Cronbach coefficient alpha (hereafter referred to as c-alpha) acted as an alternative way to investigate the degree of correlation among the set of indicators, which is the most common estimate of internal consistency of items in a model or survey (Nardo et al. 2008). Normality on data distribution is not generally an assumption needed when running c-alpha, and it is found as a cross-validation output in statistical software with NLPCAs. c-alpha is not a statistical test, but a coefficient of reliability based on the correlation between individual indicators. If the correlation is high, there is evidence that the individual indicators are measuring the same underlying construct. Therefore, a high c-alpha (or equivalently a high “reliability”) indicates that the individual indicators measure the latent phenomenon well (Nardo et al. 2008). In this second approach, Cronbach’s alpha in SPSS CATPCA is selected because it allows for the use of indicators with different scale levels (refer to Table 1 for a list of indicators of the four constructs in this study).

#### Regression

After assessing the reliability of the indicators and the internal consistency for the four constructs of Experiences, Mentoring, Awareness and Perceptions representing UREs in this study, a composite score for each construct was created. Only two of the indicators in the Perception construct needed to be reverse-scored. The composite score for each of the constructs was calculated by adding the scores of their indicators. The composite scores of each construct became the response (dependent) variables: Experience, Mentoring, Awareness and Perception. The predictor (independent) variables selected were gender (GEN), classification (CLAS), ethnicity (ETH), and first generation (FG).

A series of categorical regression models were conducted with the aforementioned response and predictor variables. The categorical regression model selected for this study is the SPSS CATREG approach, which incorporates optimal scaling and can be used when the predictor(s) and response variables are any combination of numeric, ordinal, or nominal. This type of regression with optimal scaling offers three scaling levels for each variable. Combinations of these levels can account for a wide range of nonlinear relationships and offer greater flexibility than other standard approaches such as analysis of variance or logistic regression. A *p* value of 0.05 or less was considered statistically significant.

#### Crosstabs

Results of the CATREG analysis led to the use of crosstabs when the relationship among predictor and response variables showed either statistical significance or a high beta value. The crosstabs procedure forms a two-way table and provides a variety of tests and measures of association. The structure of the table, and whether categories are ordered, determines what test or measure to use. Spearman’s rank correlation coefficient is a non-parametric measure of association between the rankings of two variables measured on *N* cases appropriate for the data structure for this study.

## Results and discussion

This study explores the UREs of STEM students and the predictors of participation based on student characteristics. As an HSI, the study site offered a unique opportunity to focus the research on LatinX students. This section presents the results of the multivariate analysis conducted and the authors’ interpretation of these findings.

As explained in the “Methods” section, a multivariate analysis was conducted on the four URE constructs explored in this study. Only the Perceptions variable presented a low c-alpha scoring of 0.285, which indicates low internal consistency of the items in the model. Indicator “P_R_Waste Time” and “P_R_ Want to Work in a Lab” were deleted from the model because of low variability and loading*.* With these two indicators deleted, the c-alpha increased to 0.680 and the component was retained. The retained indicators of “P_R Relevant,” “P_R_Only for Future Scientists,” and “P_R_Learn about Field” composed the model, and they were used in the data analysis. The other three response variables of Experiences, Mentoring, and Awareness had significantly higher c-alpha that ranged from 0.830 to 0.912. CATPCA confirmed the expected structure of the constructs to theory. All constructs had the highest c-alpha with only one dimension selected for the solution, and all component loadings in the solution were higher than 0.3, which is considered appropriate for this study (see Tables 2, 3, 4 and 5).

**Table 2:** Experiences component loadings

	Dimension 1
E_Talk_University Faculty	0.933
E_Talk_Faculty Outside	0.408
E_Work_University Faculty	0.919
E_Work_Outside	0.935
E_Aspirations_University Faculty	0.774
E_Visit University Lab	0.884
E_Visit Lab Outside	0.636

**Table 3:** Awareness component loadings

	Dimension 1
A_R_University	0.711
A_R_Department	0.760
A_R_Your Professors	0.884
A_P_ University Professors	0.801
A_P_Major Professors	0.832
A_UG R_Conferences at University	0.839
A_UG R_Conferences Dptm.	0.784
A_UG R_Outside University	0.659

Variable principal normalization

**Table 4:** Perception component loadings

	Dimension 1
P_R_Relevant	0.613
P_R_Only for Future Scientists	−0.592
P_R_Learn about Field	0.656
P_R_Skills	0.786
P_R_Lonely	−0.648

**Table 5:** Mentoring component loadings

	Dimension 1
M_Work with Faculty	0.905
M_Ask Faculty	0.831
M_Join Program	0.853

### Categorical regression analysis

For the categorical regression analysis, the variables GEN, CLAS, ETH, and FG were used as predictor variables. The response variables were Experience, Awareness, Perception, and Mentoring. To answer the research question, the categorical regression models were run using SPSS CATREG. This section will present the results of the categorical regression analysis organized by the predictor variable.

Results of the categorical regression models show that the CLAS and ETH variables were statistically significant predictors of UREs. Although, GEN and FG were not predictors of UREs at a statistically significant level of a *p* value of 0.05 or less, result of the crosstabulation analysis showed interesting relationships in the data. Limitations of the study should be considered in the interpretation of the findings. The sample size of 28 for the categorical regression analysis could be considered a small sample. Efforts were made to minimize the sample size effect by using a *p* value of 0.05 or less for statistically significant results and having an adequate response to question ratio for the regression models (five times the number of variables). Below are the results in more detail.

#### Gender

When looking at gender as a predictor of UREs, data analysis indicates that gender was not a predictor of any of the response variables in this study. However, when looking at the individual coefficient score of GEN and Perception, GEN had the second highest beta coefficient score of 0.490 in the model with a *p* value of 0.078. The beta value indicates how much change (measured by standard deviation) in the predictor variable is produced by a change in each of the response variables when others remain constant. In categorical regression, the beta value is interpreted as the difference in the predicted value of the predictor variable for each one-unit difference in each response variable when others remain constant. Given these results, a crosstab analysis was deemed appropriate to explore any emergent relationships among the variables.

Upon closer examination of the crosstab results of GEN and of the Perception variable indicators, there was information of interest reflected in the crosstab examination. Seven percent of the female respondents responded that they “somewhat agree” with the statement that research is a lonely activity in comparison with 23% of males (refer to Table 6). Negative preconceived notions about scientists and their work environment are still prevalent in STEM students. There is a need to address this misconception that might influence student career choices.

**Table 6:** P_R_Lonely * GEN Crosstabulation

P_R_Lonely		Female	Male	Gender non-conforming	Total
Somewhat disagree	Count	9	5	1	15
	% within P_R_Lonely	60.0	33.3	6.7	100.0
	% within Gender	64.3	38.5	100.0	53.6
Neither agree nor disagree	Count	4	5	0	9
	% within P_R_Lonely	44.4	55.6	0.0	100.0
	% within GEN	28.6	38.5	0.0	32.1
Somewhat agree	Count	1	3	0	4
	% within P_R_Lonely	25.0	75.0	0.0	100.0
	% within GEN	7.1	23.1	0.0	14.3
Total	Count	14	13	1	28
	% within P_R_Lonely	50.0	46.4	3.6	100.0
	% within GEN	100.0	100.0	100.0	100.0

#### Classification

The Awareness and Perception variables showed a statistical significant relationship with the predictor variable CLAS. These results are not surprising since it is expected that upper division students would have had more experiences related to research than freshmen or sophomores.

The CATREG analysis for CLAS with Awareness and Perception, produced an *F* test *p* value of 0.001 and 0.006, respectively. A crosstab analysis revealed strong correlations among 50% of the indicators in the Awareness construct and CLAS. These indicators are A_R_University, A_R_Your Professors, A_UG R_Conferences Dptm., and A_UG R_Outside University. The crosstab examination also revealed a strong correlations among student classification and the indicator P_R_ Want to Work in a Lab (refer to Table 7 for more details).

**Table 7:** CLAS statistically significant correlations with Awareness and Perception indicators

Spearman correlations	Value	Approximate significance
CLAS * A_R_University	− 0.415	0.028
CLAS * A_R_Your Professors	− 0.352	0.066
CLAS * A_UG R_Conferences Dptm.	− 0.418	0.027
CLAS * A_UG R_Outside University	− 0.400	0.035
CLAS * P_R_Only for Working in Lab	− 0.494	0.008

Again, these results are not surprising as it is expected that students’ classification will impact their awareness of scholarly activities at their institution and their perceptions on research. Of interest is that research experiences do not appear to be influenced by student classification. This is an important finding because it gives a glimpse into the status of the overall undergraduate research experiences by upper division students in the context of this study. Students are aware of research being conducted in their institution, and more particularly their departments; however, they are not engaging in these research efforts.

#### Ethnicity

Ethnicity was a predictor of students’ research experiences. Furthermore, although not statistically significant, ethnicity had a strong association with students’ perception on research. LatinX and Black students tended to talk to faculty about career aspirations much less than White students. Data also indicates that LatinX students are more likely to hold the misconception that research activities are only for future scientists.

The *F* test produced a *p* value of 0.03 when the ETH variable is paired with the Experiences variable. The CATREG for the other three constructs did not show a statistically significant *F* test value. Although ETH was not a statistically significant predictor of Perception at the *p* value of < 0.05%, it had a high beta value. This result led to a closer exploration of this association.

The ETH and Perceptions crosstab analysis for the indicator P_R_Only for Future Scientists—Q4_3 *Research is only for students that want to be scientists*—presents results worth exploring. Although results do not show a statistically significant correlation, it is important to point out that 60% of all respondents that “strongly agree” were LatinX, compared with 20% each of Black and White students. Eighty percent of the total number of students that answered “somewhat agree” were freshmen, men, and not first-generation and 20% were female, Hispanic or Latina, and first-generation (refer to Table 8 for the crosstabulation results).

**Table 8:** P_R_Only for Future Scientist * ETH Crosstabulation

P_R_Only for Future Scientist		ETH			Total
		African American	Hispanic or Latino	White	
Somewhat disagree	Count	1	4	7	12
	% within P_R_Only for Future Scientist	8.3	33.3	58.3	100.0
	% within ETH	50.0	33.3	50.0	42.9
Neither agree nor disagree	Count	0	5	5	10
	% within P_R_Only for Future Scientist	0.0	50.0	50.0	100.0
	% within ETH	0.0	41.7	35.7	35.7
Somewhat agree	Count	0	0	1	1
	% within P_R_Only for Future Scientist	0.0	0.0	100.0	100.0
	% within ETH	0.0	0.0	7.1	3.6
Strongly agree	Count	1	3	1	5
	% within P_R_Only for Future Scientist	20.0	60.0	20.0	100.0
	% within ETH	50.0	25.0	7.1	17.9
Total	Count	2	12	14	28
	% within P_R_Only for Future Scientist	7.1	42.9	50.0	100.0
	% within ETH	100.0	100.0	100.0	100.0

Other studies have suggested that being students in a STEM discipline does not necessarily imply that students hold positive images of scientists (Adedokun and Burgess 2011). Adding to this preconception (or misconception), if STEM LatinX students believe that research is only for students that want to be a scientist, it might impact their motivation to participate in UREs. Furthermore, if academic environments do not foster students’ identity development as scientists, this might also impact their level of URE participation since they do not see themselves as scientists. When undergraduate students break through the barrier between them and undergraduate research, their perceptions of science, their future careers, and the world broaden with intelligence and maturity.

#### First generation

When looking at first-generation status as a predictor of UREs, data analysis shows no statistical significance. However, crosstab analysis showed that students who self-identified as first-generation tend to be more aware of their professors’ research activity than those that are not first-generation students.

Sixty percent of first-generation students answered they are aware of their professors’ research “about half the time,” “most of the time,” or “always,” and 80% of these students were female (Table 9). By comparison, 29% of those students that are not first-generation answered this question in the same way, and of these, 50% were female. The high awareness of first-generation students relative to non-first-generation students of research performed by their professors in this study is promising. Although the results are positive, more needs to be done to translate this awareness into actual participation in research activities. Other studies report that first-generation students benefit the most when compared with non-first-generation students and other student groups in practices like undergraduate research; however, first-generation students lag behind in participation (Finley and McNair 2013; Haeger et al. 2015).

**Table 9:** A_R_Your Professor * FG Crosstabulation

A_R_Your Professors		FG		Total
		Yes	No	
Never	Count	2	7	9
	% within A_R_Your Professors	22.2	77.8	100.0
	% within FG	20.0	41.2	33.3
	% of Total	7.4	25.9%	33.3
Sometimes	Count	2	5	7
	% within A_R_Your Professors	28.6	71.4	100.0
	% within FG	20.0	29.4	25.9
	% of Total	7.4	18.5	25.9
About half the time	Count	1	3	4
	% within A_R_Your Professors	25.0	75.0	100.0
	% within FG	10.0	17.6	14.8
	% of Total	3.7	11.1	14.8
Most of the time	Count	2	0	2
	% within A_R_Your Professors	100.0	0.0	100.0
	% within First- Generation	20.0	0.0	7.4
	% of Total	7.4	0.0	7.4
Always	Count	3	2	5
	% within A_R_Your Professors	60.0	40.0	100.0
	% within FG	30.0	11.8	18.5
	% of Total	11.1	7.4	18.5
Total	Count	10	17	27
	% within A_R_Your Professors	37.0	63.0	100.0
	% within FG	100.0	100.0	100.0
	% of Total	37.0	63.0	100.0

#### A closer look at mentoring experiences

Given the low number of students reporting participation in mentoring activities, it is not surprising that data analysis did not yield any statistically significant results for the Mentoring variable. The regression analysis of student characteristics and the Mentoring variable produced low values for the adjusted *R*-square and no statistical significant result for any of the indicators. These results are not surprising given the low variability in the Mentoring indicators. In addition to the close-ended questions on student mentoring experiences, students responded to five open-ended questions. Given the importance of mentoring, more specifically faculty mentoring, in the psychosocial support and important skill development of STEM students (Winkle-Wagner et al. 2010; Collins 2013; Wilson et al. 2012), hearing from the students about their mentoring experiences was deemed important (Table 10).

**Table 10:** Students that reported active participation in mentoring (*n* = 8)

Population	Count	Research & problem solving	Academic coaching & advising	Positive attitude	Confidence course selection and career guidance
Gender/sex					
Male	4	1	2	1	2
Female	2	1			1
Undetermined	2				
Ethnicity/race					
LatinX	4	2	2		2
Black	2			1	1
Undetermined	2				
Ethnicity/race/sex					
Black female	1	N/A			
Black male	1			1	1
Latina female	1		1	1	
Latino males	3	2		2	
Undetermined	2				

Of the 35 undergraduate participants, only eight noted that they had participated in a mentoring relationship. Fifty percent of these students felt like their experiences with mentors had a significant impact. Activities of most significance included research and problem solving outside of the regular classroom, which promoted personal and academic development. Academic coaching and advising that was coupled with mentoring proved helpful in terms of general course selection. For example, one student shadowed his mentor while conducting research with doctoral students.

Five of the eight mentees also reported active participation in a mentoring relationship specifically fostered a positive attitude and perspective toward their major. The group that credited mentoring for their academic success, and described mentors as academic coaches, also fostered a sense of confidence and heightened awareness about pursuing their particular major and career pathways. One physics student paired with a chemistry professor reported changing his major as a result of the time spent with his mentor and research conducted in chemistry.

As shown in Table 10, further analysis of the responses offered identified patterns and additional insight. Of those having mentors, four were males, two females, and two records were missing gender data. All of the males gained a more positive attitude toward their majors as a result of the mentoring relationship, and the career guidance was more significant for Black males while the Latino (males) valued more the hands-on learning with problem solving and research. Similar to the Black male, the Latina female reported a more positive attitude toward major with coaching and advising as significant factors in her positive experience. The Black female was indifferent in terms of significant experiences that have had any impact on her. The other two unidentified students offered no elaboration in terms of their mentoring relationships.

The benefits of mentoring that these eight students reported are indicative of and supported by the research. While this sample of students is small, it is representative of the larger issue of mentoring in STEM (Collins 2017). For example, just as the one Black female reported no significant impact for our data, a synthesis of the literature by Ong et al. (2011) revealed that significant mentoring relationship for women of color in STEM are rare, but very beneficial when they do occur.

## Conclusions

As previous studies have shown, LatinX students participate in undergraduate research at lower rates than White or Black students (Haeger et al. 2015). This study sought to go beyond just ethnicity as a predictor of UREs participation by looking into the effect of other student characteristics on different types of UREs. Results indicate that classification and ethnicity were the strongest predictors of UREs. While first-generation status and gender were not statistically significant predictors, they showed strong association with some indicators of the Awareness and Perception constructs, respectively. This study uncovered encouraging findings about high awareness of LatinX and first-generation students on research activities at their institution.

Notwithstanding the aforementioned promising findings, this study corroborates previous findings on the low participation of undergraduate students in UREs at HSIs. Two thirds of the students that participated in this study (24 out of 35) had never participated in an URE with a faculty member. Exploring the factors contributing to this low participation rate is beyond the scope of this study; however, some insights can be derived from the findings. Misconceptions of LatinX students about research as an activity only for students that want to be scientists or who want to work alone are worth exploring as barriers of participation. The fact that only eight students reported participation in a mentoring relationship might also hinder student participation. Faculty mentors can be great resources for students to learn about research opportunities and guide students through the process of academic engagement.

The benefits of undergraduate research for underrepresented students are evident (Seymour et al. 2004; Hunter et al. 2007; Munawar 2015). It is an imperative to enhance LatinX STEM student UREs as part of the overall retention strategies of HSIs; however, the implications of these findings can transcend the context in which the study was conducted. Although, this case study focused on LatinX students in the U.S., retention of historically underrepresented students in STEM disciplines is a concern shared by many countries. The constant migration of people has shaped, and will continue to shape, the changing demographics in many regions of the world. For countries to remain competitive in the global economy, institutions of higher education need to pay closer attention to barriers that might be present for historically underrepresented students in STEM to engage in UREs. The successful recruitment, retention, and eventual success of students in STEM depend greatly on the type of pathways and support that are offered. UREs might be one of those pathways.

### Limitations and future research

Our study sample population consisted of undergraduate STEM students that participated in an undergraduate research open house event at an HSI institution. Because of the sampling method used, inherent bias in the characteristics of the participants might be present. Another consideration when looking at the results is the sample size effect. The sample size for the regression analysis with the Experience and Awareness variables is smaller than the conventional standard of at least five times the number of variables. To maximize power and minimize sample size effects, an alpha value larger or equal to 0.05 was sought. Additionally, crosstab analysis further informed the regression results.

Implications of the findings in this study point at the need to continue this research agenda. Further research is needed with a bigger sample population to further explore why awareness of research opportunities and activities of LatinX and first-generation students is not translating into actual participation in research activities with faculty at HSIs. In addition, given the potential benefits of having a faculty mentor, it is important to better understand the low participation numbers of undergraduate STEM students in these mentoring relationships. Consideration of institutional barriers to UREs participation should also be explored.

## Abbreviations

CATPCA: Categorical Principal Component Analysis
COSE: College of Science and Engineering
HSI: Hispanic-serving institution
NLPCA: Non-linear principal component analysis
PCA: Principal component analysis
SPSS: Statistical Package for the Social Sciences
STEM: Science, technology, engineering, and math
URE MAPS: Undergraduate Research Experiences: Mentoring Awareness, and Perceptions Survey
UREs: Undergraduate research experiences
